# Transcriptome Profiling Reveals Distinct Phenotype of Human Bone Marrow Mesenchymal Stem Cell-derived Hepatocyte-like cells

**DOI:** 10.7150/ijms.36255

**Published:** 2020-01-14

**Authors:** Dongyan Shi, Jiaojiao Xin, Yingyan Lu, Wenchao Ding, Jing Jiang, Qian Zhou, Suwan Sun, Beibei Guo, Xingping Zhou, Jun Li

**Affiliations:** 1 State Key Laboratory for Diagnosis and Treatment of Infectious Diseases, Collaborative Innovation Center for Diagnosis and Treatment of Infectious Diseases, The First Affiliated Hospital, Zhejiang University School of Medicine. 79 Qingchun Rd., Hangzhou, 310003. China.; 2 Clinical Medical Laboratory, Tongde Hospital of Zhejiang Province. 234 Gucui Rd., Hangzhou, 310012. China.; 3 Taizhou Central Hospital, Taizhou University Hospital. 999 Donghai Rd., Taizhou, 318000. China.

**Keywords:** hepatocyte differentiation, mesenchymal stem cell, mRNA sequencing.

## Abstract

**Background**: Human bone marrow mesenchymal stem cell-derived hepatocyte-like cells (hBMSC-HLCs) are a promising alternative for primary human hepatocytes (HHs) for treating liver disease. However, the molecular characteristics of HLCs remain unclear. Here, we aimed to clarify the transcriptome characteristics of hBMSC-HLCs for future clinical application.

**Materials and Methods**: hBMSCs were isolated from the bone marrow of healthy volunteers and differentiated into hepatocytes. mRNA sequencing was used in the transcriptome profiling of hBMSC-HLCs, with hBMSCs and HHs as controls.

**Results**: hBMSC-HLCs exhibited a polygonal morphology, glycogen accumulation and albumin expression. A total of 630 upregulated and 1082 downregulated genes were observed in hBMSC-HLCs and HHs compared with undifferentiated hBMSCs. The upregulated genes were mainly involved in hepatic metabolism and inflammatory and immune responses. The downregulated genes were mainly associated with stem cell characteristics (multipotent differentiation, cell cycle regulation, etc.). Confirmatory qRT-PCR of 9 upregulated and 9 downregulated genes with log2 fold changes > 5 showed similar results. *In vivo* transdifferentiation of hBMSCs in pigs with fulminant hepatic failure confirmed the similarly upregulated expression of 5 hepatogenic genes (*TDO2, HP, SERPINA3, LBP* and* SAA1*), showing a 150-fold change in liver tissues at 7 days after hBMSC transplantation. These 5 genes mainly contributed to liver metabolism and inflammation.

**Conclusion**: hBMSC-HLCs possess a hepatic transcriptome profile and express hepatic-specific genes *in vitro* and *in vivo*, which might be useful for future clinical applications. The five upregulated genes identified herein could be potential biomarkers for the characterization of hBMSC-HLCs.

## Introduction

Bone marrow mesenchymal stem cell (BMSC)-dervied hepatocyte-like cells (BMSC-HLCs) have the potential to overcome the limitations of primary human hepatocytes (HHs) for clinical application in treating liver disease and for drug development 1, 2. These BMSC-HLCs can restore liver functions and are engrafted predominantly in the periportal portion of the liver lobule, as shown in small- and large- animal models of acute liver failure and liver regeneration 3-6. Autologous BMSC transplantation in patients with end-stage liver failure showed significant improvement in liver functions, including serum albumin levels, Child-Pugh scores and model for end-stage liver disease scores, and the damaged liver tissues were repaired by BMSC-HLCs 7, 8.

Although several strategies are available for generating BMSC-HLCs, these cells are still less effective than HHs in repopulating the liver, and the transcriptome mechanism is still unclear 2, 9-11. The currently available characterizations of these differentiated hepatocytes are usually based on a select set of gene markers. Microarray analysis of gene expression during differentiation is the most commonly used method of studying the underlying mechanism, especially for the hepatocyte differentiation of human embryonic stem cells (ESCs) or human-induced pluripotent stem cells (hiPSCs). The *in vitro* differentiation of hESCs to a hepatic lineage involves a sequential epithelial-mesenchymal-epithelial transition (EMT-MET) and the participation of differentially expressed genes involved in proliferation, extracellular matrix-related functions, hepatic metabolism and normal liver functions 10, 12, 13. During differentiation, the TGFβ-SNAl1 pathway is believed to play a critical role in the EMT phase; however, little is known about the transcriptional regulatory network during the MET phase 13. Gene microarray analysis of human adipose tissue-derived stromal cell (ATSC)-differentiated hepatocyte-like cells revealed a complex interplay between cell receptors, signaling pathways, and transcription factors that allow tissue cross-lineage conversion during differentiation and the subtle regulation of the canonical pathways, BMP, WNT and TGFβ may be important in the MET process 11. Our previous study using a cytokine array found that the differentiation of human BMSCs into hepatocytes is associated with the expression of TIMP-4 and FST 14.

Herein, we report a gradual loss of pluripotency and gain of hepatic characteristics during human BMSC (hBMSC) hepatic differentiation using a whole-genome mRNA sequencing (mRNA-seq) analysis and compared the results with the mRNA-seq data for HHs. Finally, the five genes (*TDO2, HP, SERPINA3, LBP* and* SAA1*) validated in hBMSC transplantation with a fulminant hepatic failure (FHF) model in pigs could be potential biomarkers for the characterization of hBMSC-HLCs.

## Materials and Methods

### Isolation, culture and phenotypic identification of hBMSCs

Human BMSCs were isolated from the bone marrow of healthy volunteers by Ficoll-Paque (GE Healthcare, Uppsala, Sweden) density-gradient centrifugation and cultured with Dulbecco's modified Eagle's medium supplemented with 10% fetal bovine serum (Invitrogen, Carlsbad, CA) at 37°C in 5% CO_2_ 6. Signed informed consent was obtained and approved by the Ethics Committee of the First Affiliated Hospital, Zhejiang University School of Medicine in fulfillment of the principles of the Declaration of Helsinki. Cells during passages 3-5 were used for the experiments.

The phenotypic identification of cultured hBMSCs was performed by standard flow cytometry methods with antibodies against human CD45 (Abcam, Cambridge, UK), CD34, CD29, and CD90 (all from BD Biosciences, CA, USA), as described in our previous work 6.

### Osteogenic, adipogenic and hepatogenic differentiation of hBMSCs

To detect the multilineage differentiation potential of hBMSCs, the cells were cultured separately in a commercially available osteogenic and adipogenic differentiation medium (both from Cyagen Biosciences, CA, USA). On day 21, the cells that underwent adipogenic induction were stained with Oil red O for lipid droplets. On day 28, the cells that underwent osteogenic induction were stained with alizarin red for calcium compounds.

Hepatogenic differentiation was performed by using a two-step protocol 6. First, hBMSCs were cultured with basal medium [Iscove's Modified Dulbecco's Medium (Invitrogen, CA, USA) supplemented with 100 ng/ml recombinant human hepatocyte growth factor (HGF; PeproTech, NJ, USA), 20 µg/ml dexamethasone (DEX; Sigma, MA, USA), 1% penicillin-streptomycin (Invitrogen, CA, USA), 1% HEPES buffer solution (Invitrogen, CA, USA), and 1% Insulin-Transferrin-Selenium premix (ITS) +1 Liquid Media Supplement (100×, Sigma, MA, USA)] for two weeks. Then, the medium was changed to maturation medium [basal medium supplemented with 20 ng/ml recombinant human oncostatin-M (OSM; PeproTech, NJ, USA)] for an additional week.

After 20 days of induction, the cells were characterized for hepatic function with immunohistochemistry (IHC) staining for albumin (ALB) and periodic acid-Schiff (PAS) staining for glycogen accumulation. Reverse transcription-polymerase chain reaction (RT-PCR) was performed to detect the hepatic-specific genes [*ALB*, α-fetoprotein (*AFP*), tryptophan 2,3-dioxygenase (*TDO2*) and tyrosine aminotransferase (*TAT*)] on days 0 (D0), 10 (D10) and 20 (D20) after hepatogenic differentiation. The primer sequences for the above genes are provided in Table 1. Glyceraldehyde 3-phosphate dehydrogenase (*GAPDH*) was used as an internal control. HHs, used as a positive control, were isolated from human liver tissue specimens that were harvested from therapeutic partial hepatectomies performed on three male patients with hepatic haemangioma (one was 46 years old, and the other two were 70 years old) who exhibited no sign of hepatic dysfunction, hepatitis virus infection or hepatic cancer. Signed informed consent was obtained and approved by the Ethics Committee of the First Affiliated Hospital, Zhejiang University School of Medicine. The isolation of HHs was performed as previously described 15, 16.

### Total RNA extraction and mRNA sequencing

Cells were harvested on D0, D10 and D20 (four samples per group). Three samples of freshly isolated HHs, used as a positive control, were immediately subjected to RNA extraction after isolation. Total RNA extraction was performed using TRIzol reagent (Invitrogen, CA, USA). A sequencing library was then prepared using the TruSeq™ RNA Sample Preparation Kit (Illumina, CA, USA), including adapter ligation, reverse transcription, PCR amplification and pooled gel purification steps. The pooled library consisted of sequences with lengths of approximately 250 nucleotides. The library was sequenced using the HiSeq 2000 sequencing system (Illumina, CA, USA). The average number of sequencing reads was approximately 22 million per RNA sample.

### Gene expression analysis of mRNA-seq data

The sequencing reads from mRNA-seq were mapped to hg38 (Ensembl GRCh38 release 78) using STAR (v2.4.0), which is an ultrafast universal RNA-seq aligner. All parameters were set to the default values, except for the allowed maximum mismatch, which was set to 5% for each read, and the output of the BAM files, which were sorted by coordinate. The transcripts were assembled de novo using Cufflinks (v2.2.1), and the novel built GTF file was merged with the GTF file of hg38. With the merged GTF file, the abundance in units of fragments per kb of exon per million mapped reads (FPKM) of each transcript was estimated using the cuffquant and cuffnorm tools within the Cufflinks suite. The differential expression of each transcript and each gene was analyzed using cuffdiff, which is also a component of Cufflinks, and further analysis was performed with the cummeRbund package (v2.12.0) in R (v3.2.3). Significantly differentially expressed genes (DEGs) were selected with *q-*value < 0.05.

### Functional clustering of the DEGs

The significantly upregulated or downregulated DEGs were submitted to the Database for Annotation, Visualization and Integrated Discovery (DAVID, v6.7) for Gene Ontology (GO) enrichment analyses. The enriched GO terms with a false discovery rate (FDR) < 0.05 were considered significant and further subjected to a cluster analysis package in DAVID.

### Selection of representative DEGs for validation

The top 9 up- and 9 downregulated DEGs with log2 fold changes of D0 and D20 > 5 times were selected for further *in vitro* and *in vivo* validation. The functional consequences of these DEGs were interpreted by the gene set linkage analysis (GSLA) 5.

### Verification of the mRNA expression levels of 18 DEGs by quantitative real-time RT-PCR (qRT-PCR)

The mRNA expression levels of the above 18 DEGs (for primers see Table 1) were verified in three additional independent hepatogenic differentiation experiments via qRT-PCR. The target genes were assayed in triplicate on each plate. *GAPDH* was used as an internal control gene to normalize and evaluate each target gene on the same plate for data comparison.

### *In vivo* validation of the 18 DEGs via mRNA-seq

The hBMSC transplantation model in pigs with FHF was established according to our previous study 5. Briefly, FHF was induced in male Chinese experimental miniature pigs (weighing 8-10 kg) by the intraperitoneal injection of D-galactosamine at a dose of 3.0 g/kg body weight. Simultaneously, hBMSCs (3 × 10^6^ cells/kg body weight) were transplanted into the FHF pigs via the intraportal vein under B-ultrasound guidance (T group). The control (C) group was transplanted with an equal volume of normal saline without cells. Liver tissues collected before FHF induction (D0), at three days after FHF induction in the C group when the pigs died (C-D3), and at three (T-D3) and seven (T-D7) days after hBMSC transplantation in the T group were subjected to hematoxylin and eosin (HE) staining and IHC staining with human-specific antibodies against CD90, CD29, ALB and HSA. The D0, C-D3 and T-D7 samples were analyzed with mRNA-seq (n = 2/group). The gene expression levels of the 18 DEGs were detected in mRNA-seq data. All animal experiments were approved by the Animal Care Ethics Committee of the First Affiliated Hospital, Zhejiang University School of Medicine, and all animals received humane care according to the criteria of the Guide for the Care and Use of Laboratory Animals.

### Statistical analyses

All statistical analyses were performed with the R software package. The significant DEGs and transcripts were analyzed using the cuffdiff and cummeRbund packages. An unsupervised hierarchical clustering of the samples from different groups with significant DEGs was performed using the pheatmap package. The DEGs validated via qRT-PCR were identified using the stats package in R software. A *P*-value of < 0.05 was considered to indicate significant differences.

## Results

### Characteristics of hBMSCs and hBMSC-HLCs

**Figure 1:**
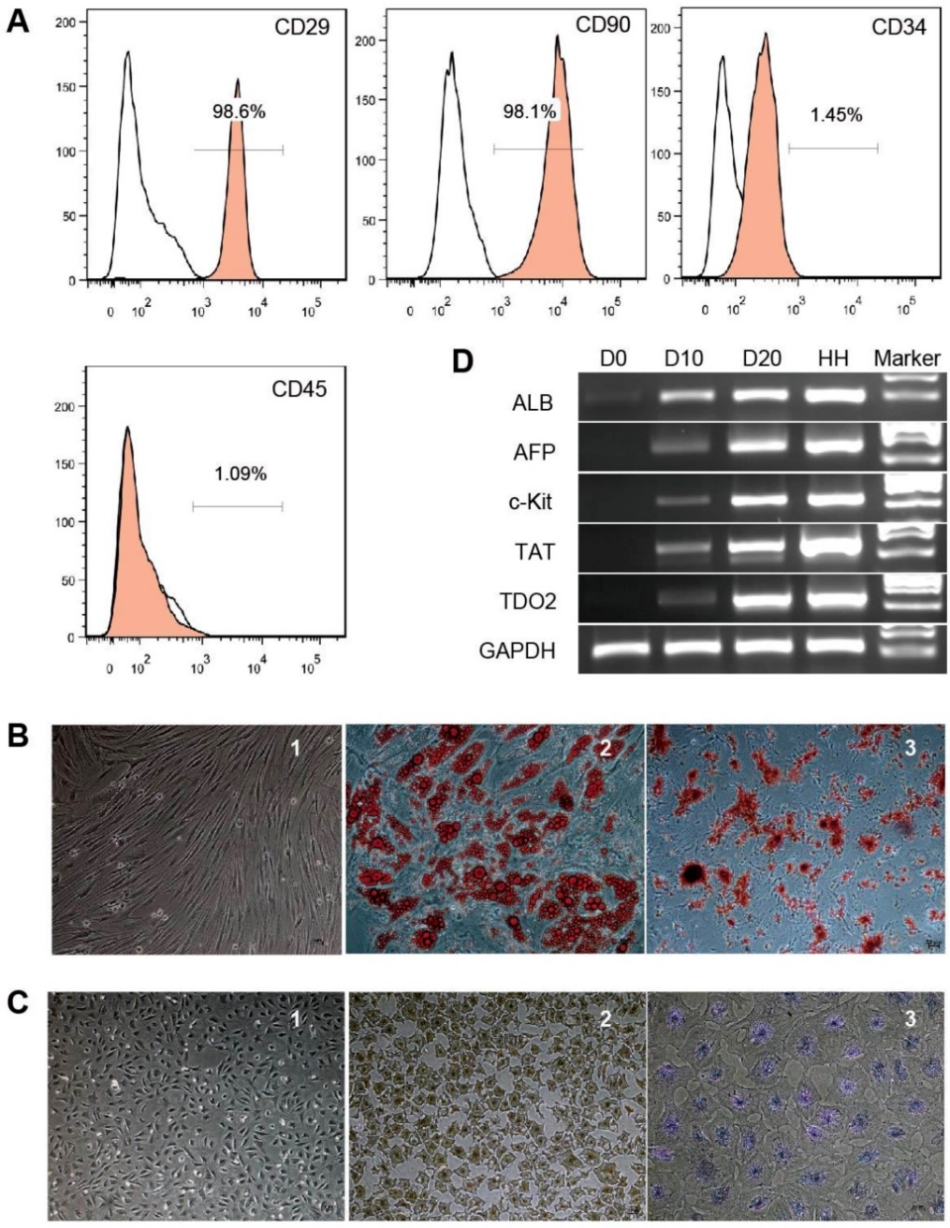
** Characterization of hBMSCs and hBMSC-HLCs.** Cultured hBMSCs were positive for CD29 and CD90 but negative for CD34 and CD45 (A). hBMSCs exhibited a fibroblast-like morphology (B-1) and differentiated into adipocytes (B-2) and osteocytes (B-3); ×10, ×20 and ×10 magnification. After 20 days of hepatogenic induction, the cells exhibited a polygonal morphology (C-1) and were positive for immunohistochemical staining for ALB (C-2) and PAS staining for glycogen accumulation (C-3); ×4, ×4 and ×10 magnification. (D) RT-PCR of hepatocyte-specific genes. The lanes, from left to right, represent undifferentiated hBMSCs (D0), hepatogenic differentiation on day 10 (D10) and day 20 (D20), primary human hepatocytes (HHs), and the marker. ALB, albumin; AFP, α-fetoprotein; TAT, tyrosine aminotransferase; TDO2, tryptophan 2,3-dioxygenase; GAPDH, glyceraldehyde phosphate dehydrogenase.

**Figure 2:**
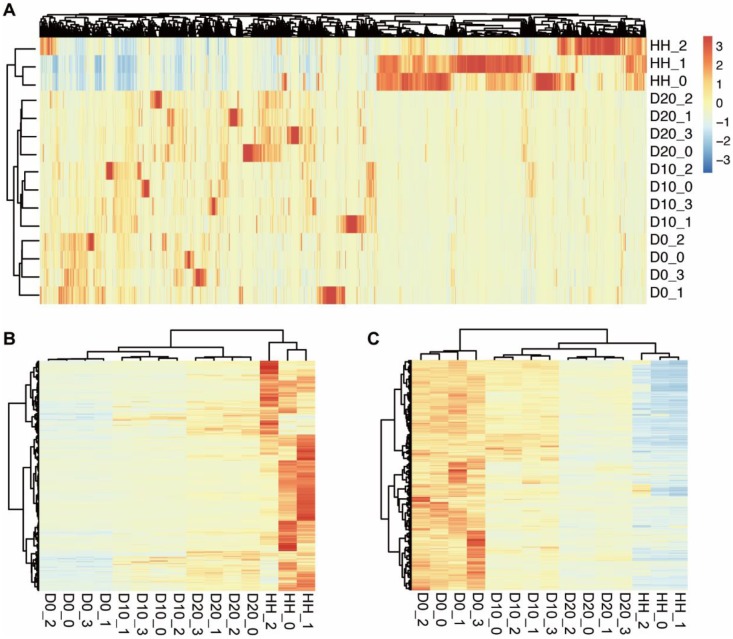
** The clustering results of samples at different stages of hepatogenic differentiation.** (A) The clustering results determined by 38411 genes expressed in at least one sample. The samples are labeled as follows: D0, undifferentiated hBMSCs; D10, cells after 10 days of hepatogenic differentiation; D20, after 20 days of differentiation; HHs, primary human hepatocytes. (B) The clustering result based on the 630 upregulated DEGs between D0 and D20/HH. (C) The clustering result based on the 1082 downregulated DEGs.

**Fig 3:**
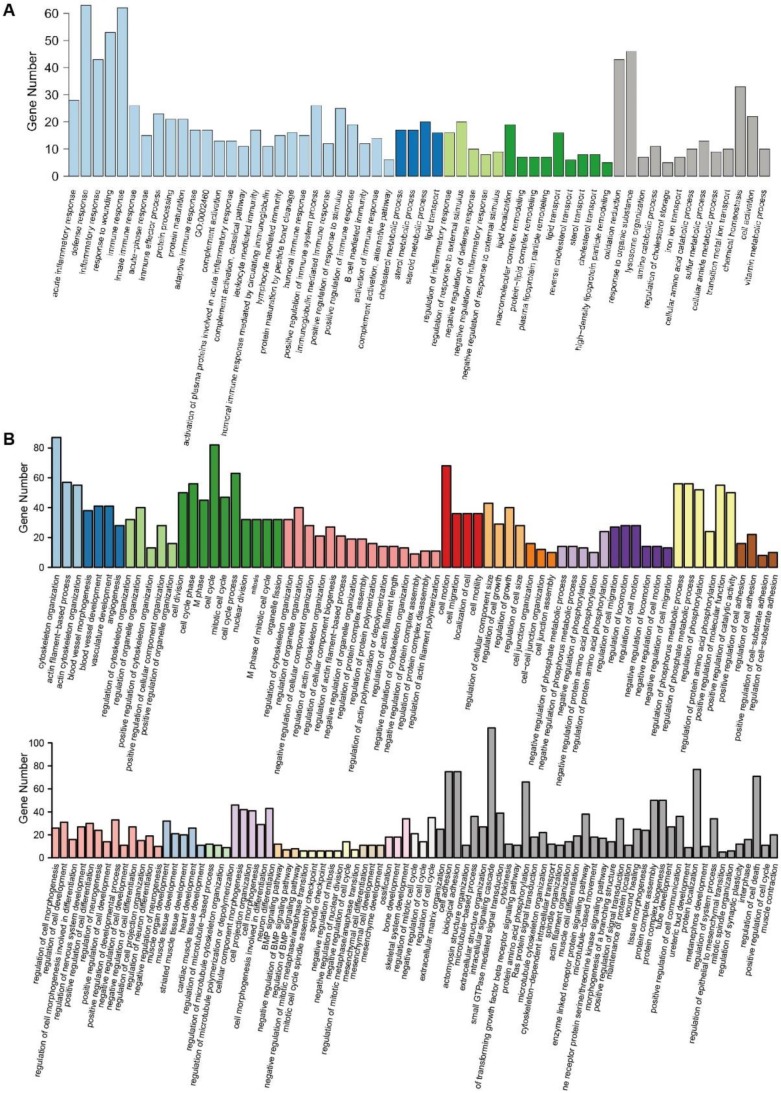
** GO enrichment analysis of DEGs.** (A) The 58 GO terms of 630 upregulated DEGs were clustered into 4 clusters with 13 individual terms. (B) The 154 GO terms of 1082 downregulated DEGs were clustered into 21 clusters with 40 individual terms.

Phenotypic analysis of hBMSCs by flow cytometry showed that the cells were positive for CD29 and CD90 (98.6% and 98.1%, respectively) but negative for CD34 and CD45 (1.45% and 1.09%, respectively) (Fig. 1A). The cells showed a fibroblast-like morphology (Fig. 1B-1). After 21 days of adipogenic induction, lipid droplets accumulated in the differentiated adipocytes as detected by Oil red O staining (Fig. 1B-2). Alizarin red staining showed calcium deposits in the differentiated osteocytes on day 28 of osteogenic induction (Fig. 1B-3). These results indicate that the cells used in this study possessed the classical phenotype and multipotent stem cell characteristics of hBMSCs.

hBMSC-HLCs exhibited a polygonal morphology with a low cytoplasm/nucleus ratio after 20 days of hepatogenic induction (Fig. 1C-1). IHC staining showed positive results for ALB (Fig. 1C-2), and the differentiated cells displayed the ability to store glycogen, as indicated by positive PAS staining (Fig. 1C-3), whereas hBMSCs were negative for both markers. RT-PCR showed that the expression levels of liver-specific genes (*ALB, AFP, TAT* and *TDO2*) increased from D10 to D20 (Fig. 1D).

### Transcriptome profiling of hBMSC-HLCs

To investigate the differences in transcriptome profiling of hBMSCs and hBMSC-HLCs, we compared the transcriptomes of undifferentiated hBMSCs (D0), hBMSC-HLCs (D10, D20) and HHs via mRNA-seq. Gene expression analysis showed that the number of sequencing paired-end reads for each sample ranged from 15.3 million to 27.9 million, and an average of 88.5% of reads from each sample were uniquely mapped to the human genome. In total, 29783, 31039, 31980 and 28539 genes were identified in the samples from the D0, D10, D20 and HH groups, respectively. Detailed information for each sample is given in Table 2. A total of 15453 genes were detected in all samples, and 38411 genes were detected in at least one sample, which was used for further analysis. Clustering analysis based on the expression of these genes was able to distinguish different stages of hepatogenic differentiation (Fig. 2A). The transcriptomes of D10 and D20 were more similar than that of D0, informing the transformation of cells under hepatogenic induction.

### Analysis of DEGs and the biological function of hBMSC-HLCs

To reveal the biological characteristics of hBMSC-HLCs, significant DEGs between different groups were detected by Cufflinks. The results showed that 2347 genes were differentially expressed between D0 and D10, 3207 between D0 and D20 and 9130 between D0 and HH groups. A total of 630 genes were significantly upregulated in both the D20 and HH groups, and 1082 were significantly downregulated. Clustering analysis using the upregulated and downregulated DEGs showed an increasing trend in transcriptomic similarity from D0 to D10, D20 and HH groups (Fig. 2B, 2C). The gene expression levels in D10 and D20 were more similar to those in the HH group. These changes in the transcriptomes inferred that after hepatogenic induction, the cells were transformed from hBMSCs to hepatocytes.

Further GO enrichment analysis confirmed these results. In total, 58 GO terms were identified with 630 upregulated DEGs. These terms were clustered into 4 clusters, while 13 terms did not belong to any of the clusters. Cluster 1 included the biological processes of inflammatory and immune responses, cluster 2 was related to sterol metabolism, cluster 3 was related to responses to stimulus, and cluster 4 was related to lipid transport. These processes were important functions of hepatocytes in the liver (Fig. 3A). There were 154 GO terms identified with 1082 downregulated DEGs. These terms were clustered into 21 clusters with 40 individual terms (Fig. 3B). In total, most of the clusters included the biological processes that reflected the characteristics of stem cells, such as the organization and remolding of the cytoskeleton, the regulation of the cell cycle and migration, and the multipotent differentiation of blood vessels, muscle, neuron, and heart.

### Representative DEGs and functional annotation

**Figure 4:**
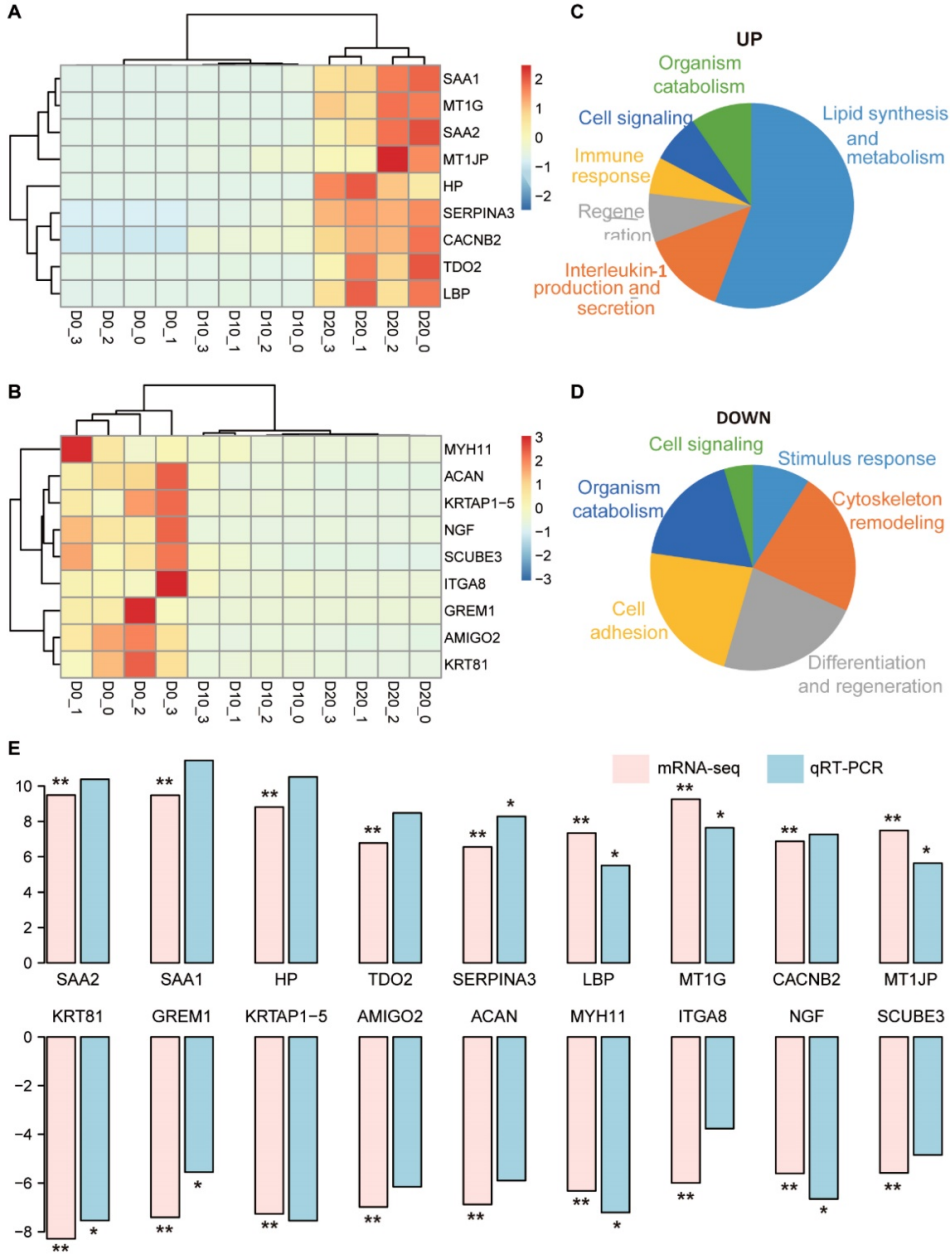
** Functional annotation of 18 representative DEGs and validation via qRT-PCR.** (A, B) The clustering results of the top 9 upregulated (A) and 9 downregulated (B) DEGs. (C, D) Biological processes related to the upregulated (C) and downregulated (D) DEGs. (E) Comparison of the log2 fold changes of the 18 DEGs in mRNA-seq analysis and qRT-PCR validation. The significance levels were compared between D20 and D0; * indicates *P*-value < 0.05, ** indicates *P*-value < 0.01.

Representative DEGs with log2 fold changes > 5 between D0 and D20 were selected for further analysis and validation, including 9 upregulated DEGs (*SAA1*, *SAA2*,* MT1G, HP, MT1JP, LBP, CACNB2, TDO2* and *SERPINA3*) and 9 downregulated DEGs (*KRT81, GREM1, KRTAP1-5, AMIGO2, ACAN, MYH11, ITGA8, NGF* and* SCUBE3*). Clustering analysis using the upregulated DEGs could distinguish D20 from D10 and D0 (Fig. 4A), and the results from downregulated DEGs distinguished D0 from D10 and D20 (Fig. 4B). The functional consequences of the 9 upregulated DEGs enriched in 50 GO terms were interpreted by GSLA. A total of 29 terms were involved in the regulation of lipid synthesis and metabolism, which was one of the most important functions of hepatocytes (Fig. 4C). Seven terms were related to the regulation of interleukin-1 (IL-1) production and secretion, suggesting that IL-1 signaling may play an important role in hepatogenic differentiation. Analysis of the 9 downregulated DEGs showed that these genes contributed to the regulation of cytoskeletal remodeling, differentiation and regeneration and cell adhesion (Fig. 4D).

### QRT-PCR validation of the representative DEGs *in vitro*

The expression levels of 18 representative DEGs were validated in three additional hepatogenic differentiation samples via qRT-PCR. The results of qRT-PCR showed complete consistency in the expression trend from D0 to D20 with that of mRNA-seq (Fig. 4E). Specifically, the gene expression levels of *SAA1*, *SAA2*, *HP*, *TDO2* and *SERPINA3*, which were biased expressed in the liver, increased > 8-times log2 fold change on D20 compared to D0: *SAA1* (11.44), *SAA2* (10.38), *HP* (10.52), *TDO2* (8.48), and *SERPINA3* (8.28).

### Validation of the 18 DEGs in hBMSC-transplanted FHF pigs

**Figure 5:**
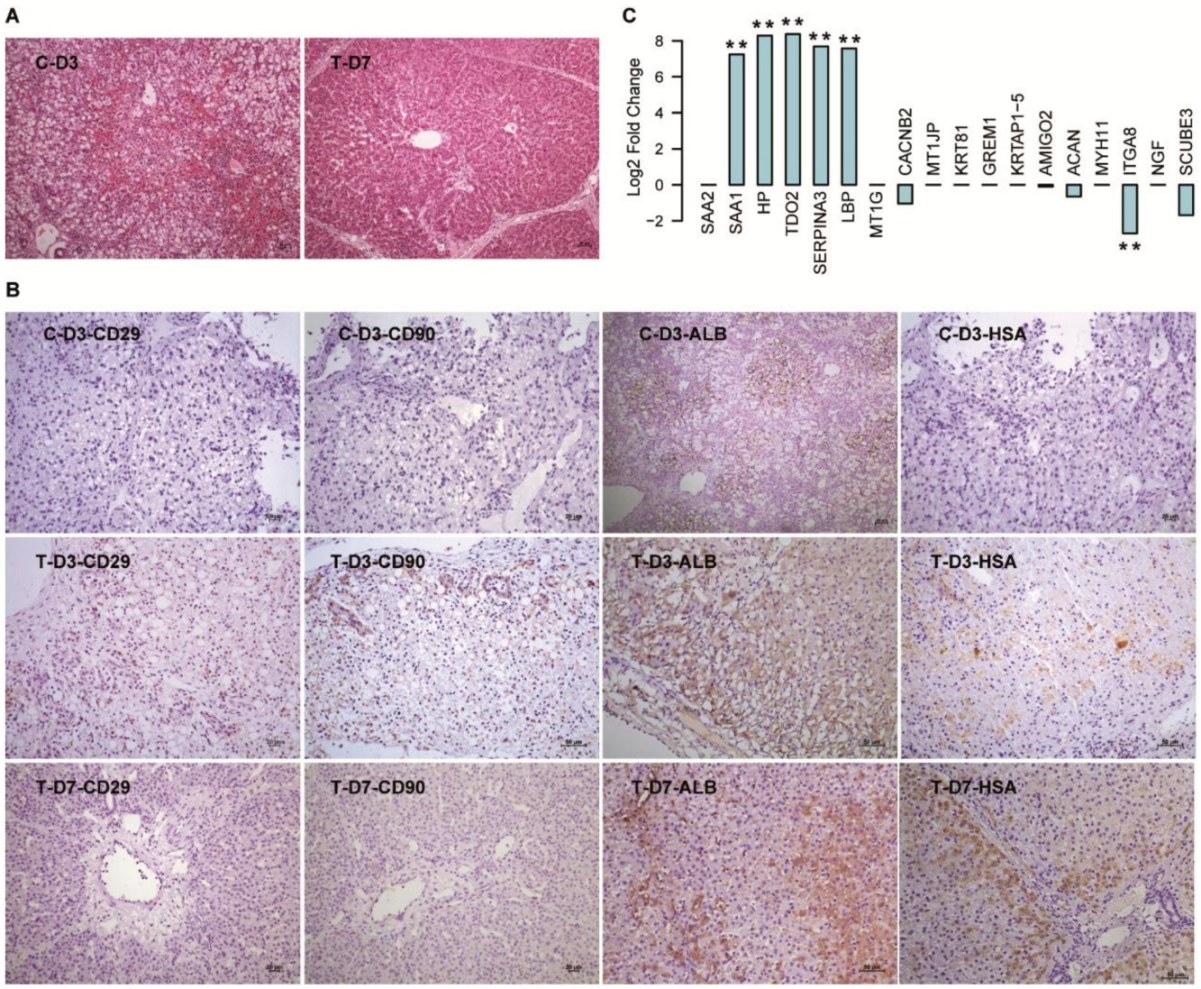
** Validation of the 18 DEGs in hBMSC-transplanted FHF pig models.** (A) HE staining of liver tissues harvested from FHF pigs that died at D3 (C-D3) and seven days after hBMSC transplantation (T-D7). (B) IHC staining of human-specific antibodies in liver tissues harvested at D3 in the C group, and at D3 and D7 in the T group. (C) Log2 fold changes in the 18 DEGs in the mRNA-seq data of liver tissues in T-D7 compared to C-D3. ** indicates *P*-value < 0.01.

Consistent with our previous research 5, the FHF pigs were rescued by hBMSC transplantation in the T group, and the pigs in the C group all died within three days. HE staining of the deceased pig liver tissue in the C group showed a typical FHF histology with extensive hepatic necrosis and hemorrhage, while the damaged liver structure showed notable repair on D7 in the T group (Fig. 5A). IHC staining with human-specific antibodies showed that there were undifferentiated hBMSCs (CD90^+^ and CD29^+^) and hBMSC-HLCs (ALB^+^ and HSA^+^) in the liver tissue from T-D3, while only hBMSC-HLCs were detected in T-D7 (Fig. 5B), indicating that the transplanted hBMSCs initiated hepatic transdifferentiation within three days and were almost finished within seven days. All these markers showed negative staining in liver tissues from C-D3. We then detected the gene expression of the 18 DEGs in the mRNA-seq data of liver tissues harvested from T-D7 and C-D3. The results showed that 5/9 upregulated DEGs were also significantly elevated in T-D7 compared to C-D3 [*TDO2* (331-fold), *HP* (312-fold), *SERPINA3* (207-fold),* LBP* (191-fold) and *SAA1* (152-fold)], with one DEG showing the opposite trend [*CACNB2* (decreased 2.06-fold)] and the other 3 DEGs (*SAA2, MT1G* and *MT1JP*) were not significantly changed (Fig. 5C). Among the 9 downregulated DEGs, only 4 genes showed a slight downregulation in T-D7. This consistency, especially with the upregulated DEGs, indicated that the hepatocytes differentiated from hBMSCs *in vitro* were similar to those developed *in vivo*.

## Discussion

The use of stem cells to generate hepatocytes with defined factors provides a new strategy for treating liver disease and for performing *in vitro* pharmacological and toxicological studies. Various reports have shown the potential of hBMSCs to differentiate toward endodermal lineages *in vitro* and *in vivo* 9. In this study, we achieved the hepatogenic conversion of hBMSCs using a two-step protocol with the sequential addition of growth factors. Under induction, the fibroblast-like hBMSCs differentiated into polygonal cells, which acquired specific liver functions, as shown by the expression of hepatic-specific genes and proteins and the accumulation of glycogen in these cells.

According to the hepatogenic induction system *in vitro*, the differentiated hepatocytes were immature, and there were drastic differences in the transcriptome profiling between hBMSC-HLCs (D20) and HHs, indicating that these cells are hardly fit for clinical applications, which requires further improvement. Therefore, we focused on the transcription changes from D0 to D20 and found that the direction of hepatic transformation during induction is clearly precise, as shown by the clustering analysis of mRNA-seq data, especially by the downregulated DEGs (Fig. 2). Further functional analysis showed that the upregulated DEGs were involved in normal liver functions, such as lipid metabolism, synthesis and transportation and the immune response, while the downregulated DEGs were associated with the functions of stem cells, such as proliferation, multiple differentiation potential, reconstruction and function of the extracellular matrix. These results are in agreement with previous studies of hepatic differentiation from hESCs, hiPSCs and hATSCs 10, 12, 17 and inferred hepatic conversion through the regulation of molecular pathways that control the lineage commitment of the MET process 11-13, 18, 19.

Based on the rationale that the identification of suboptimal gene networks represents a tractable target for improving cell phenotype, the major hurdle in the path to stem cell-derived hepatocyte-like cells is the control mechanisms for the above biological processes; thus, adjusting the involved gene expression levels to those of HHs should be helpful for generating functional hBMSC-HLCs. Interestingly, 5/9 upregulated DEGs (*TDO2, HP, SERPINA3, LBP* and* SAA1*) exhibited the same expression trend in our in-house hBMSC-transplanted FHF pigs, in which hBMSCs had differentiated into hepatocytes within 7 days (Fig. 5). These 5 genes were highly expressed in primary hepatocytes in our study (Supplementary Fig. 1) and have been reported to be restricted or show biased expression in the liver and contribute to the liver functions of lipid synthesis, metabolism, IL-1 production and secretion regulation 20-24. These results indicated that hBMSC-HLCs generated* in vitro* had gene expression patterns similar to those of hBMSC-HLCs developed *in vivo*.

In conclusion, mRNA transcriptome profiling indicated the mechanical direction of hBMSC differentiation into hepatocyte-like cells. Considering the immature HLCs differentiated *in vitro*, the above 5 DEGs may not be hepatocyte-specific, but they could be potential biomarkers for the characterization of HLCs, which should be validated during hepatic differentiation from other stem cell sources in the future.

## Supplementary Material

Supplementary figure.

## Figures and Tables

**Table 1:** Primers used in this study.

Gene	Abbreviation	NCBI accession number	Length	Primer
Albumin	ALB	NM_000477.5	164	ctgagcaaaggcaatcaaca cacagtctgctgaggttgga
α-Fetoprotein	AFP	NM_001134.1	248	agcttggtggtggatgaaac ccctcttcagcaaagcagac
c-kit receptor tyrosine kinase	c-Kit	NC_000004.11	218	gtgaccaacatggagtcgtg tgcttcacagaagaccatgc
Tyrosine aminotransferase	TAT	NM_000353.1	236	tagcttctaggggtgcctca agccattgtggacaacatga
Tryptophan 2,3-dioxygenase	TDO2	NM_005651.3	222	gtgcatccgagaaacaacct gggaactacctgcatttgga
Glyceraldehyde 3-phosphate dehydrogenase	GAPDH	NM_002046.3	113	ctctctgctcctcctgttcg acgaccaaatccgttgactc
Serum amyloid A2	SAA2	NM_001127380.2	245	tctgaaggctcagtgtggtg cttctcctgcaaccaccttc
Serum amyloid A1	SAA1	NM_000331.5	200	cacagatcaggtgaggagca gaccaaggagcagaaaacca
Metallothionein 1G	MT1G	NM_001301267.1	232	tcctgcaagtgcaaagagtg ggaatgtagcaaaggggtca
Haptoglobin	HP	NM_001126102.2	214	taacccacacgccctacttc taacccacacgccctacttc
Metallothionein 1J, pseudogene	MT1JP	NR_036677.1	207	ctcgaaatggaccccaacta agagctgttcccacatcagg
Lipopolysaccharide binding protein	LBP	NM_004139.4	232	tggaagcgctcctcaactat ctggaaatgcaacaagcaga
Calcium voltage-gated channel auxiliary subunit beta 2	CACNB2	NM_000724.3	227	gtaccttccatgcgaccagt tccgctaagcttgaccttgt
Serpin family A member 3	SERPINA3	NM_001085.4	163	agcagtggggctctcagtaa ccctgtgcatgtgagagcta
Keratin 81	KRT81	NM_002281.3	163	taggcaccccaactcaagtc aagtgggggatcacacagag
Gremlin 1, DAN family BMP antagonist	GREM1	NM_001191322.1	194	gctctggcattcagagaacc aaattcgcctagcgtgagaa
Keratin associated protein 1-5	KRTAP1-5	NM_031957.1	249	atgctacaaagccacctgct gttgggtgatgagcttccat
Adhesion molecule with Ig like domain 2	AMIGO2	NM_001143668.1	172	tcgtttgcaaagctgaacac gcagaagcacttccagaacc
Aggrecan	ACAN	NM_001135.3	189	acagctggggacattagtgg gtggaatgcagaggtggttt
Myosin heavy chain 11	MYH11	NM_001040113.1	182	ggaggatgagatcctggtca ttagccgcacttccagttct
Integrin subunit alpha 8	ITGA8	NM_001291494.1	218	cacattctggtggactgtgg aatcccttgttgttgcgttc
Nerve growth factor	NGF	NM_002506.2	239	tcagcattcccttgacactg tgctcctgtgagtcctgttg
Signal peptide, CUB domain and EGF like domain containing 3	SCUBE3	NM_001303136.1	218	gattgcacagagccactgaa gagttggtgctgttcccatt

**Table 2:** Qualitative analysis results of transcriptomes at different differentiation stages.

Sample	Total reads	Uniquely mapped reads	Uniquely mapped rate %	Number of genes
D0_0	20540108	19300051	93.96%	23732
D0_1	15302865	14144013	92.43%	24937
D0_2	20006894	17471349	87.33%	23471
D0_3	19638793	18352256	93.45%	23540
D10_0	22984560	21546799	93.74%	24678
D10_1	18807159	17565119	93.40%	26142
D10_2	20801499	19270370	92.64%	24416
D10_3	21656601	20417941	94.28%	24365
D20_0	23909425	21703501	90.77%	26539
D20_1	24977495	22871338	91.57%	26234
D20_2	22120789	20228016	91.44%	25192
D20_3	23518297	21692270	92.24%	26163
HH_0	27946164	20538064	73.49%	24563
HH_1	25101600	17552544	69.93%	23122
HH_2	22656382	17324623	76.47%	24375

## Abbreviations

ALB: albumin
ATSC: adipose tissue-derived stromal cell
BMSC: bone marrow mesenchymal stem cell
DEG: significantly differentially expressed gene
EMT-MET: epithelial-mesenchymal-epithelial transition
ESC: human embryonic stem cell
FDR: false discovery rate
FHF: fulminant hepatic failure
FPKM: fragments per kb of exon per million mapped reads
GO: Gene Ontology
GSLA: gene set linkage analysis
hBMSC-HLCs: human bone marrow mesenchymal stem cell-dervied hepatocyte-like cells
HH: primary human hepatocyte
hiPSC: human-induced pluripotent stem cell
IHC: immunohistochemistry staining
IL-1: interleukin-1
mRNA-seq: mRNA sequencing
PAS: periodic acid-Schiff
qRT-PCR: quantitative real-time reverse transcription-polymerase chain reaction
