# Supplementary material for: Transcriptome Profiling Reveals Distinct Phenotype of Human Bone Marrow Mesenchymal Stem Cell-derived Hepatocyte-like cells
Source: Int J Med Sci. 2020 Jan 14;17(2):263–73. doi: 10.7150/ijms.36255 (PMC6990879; doi:10.7150/ijms.36255)

Suppl. Fig. 1. Gene expression of *SAA2*, *SAA1*, *HP*, *LBP* and *TDO2* at different stages of hepatogenic differentiation in mRNA-seq data. The samples are labeled as follows: D0, undifferentiated hBMSCs; D10, cells after 10 days of hepatogenic differentiation; D20, cells after 20 days of differentiation; HHs, primary human hepatocytes.

Supplemental Figure 1

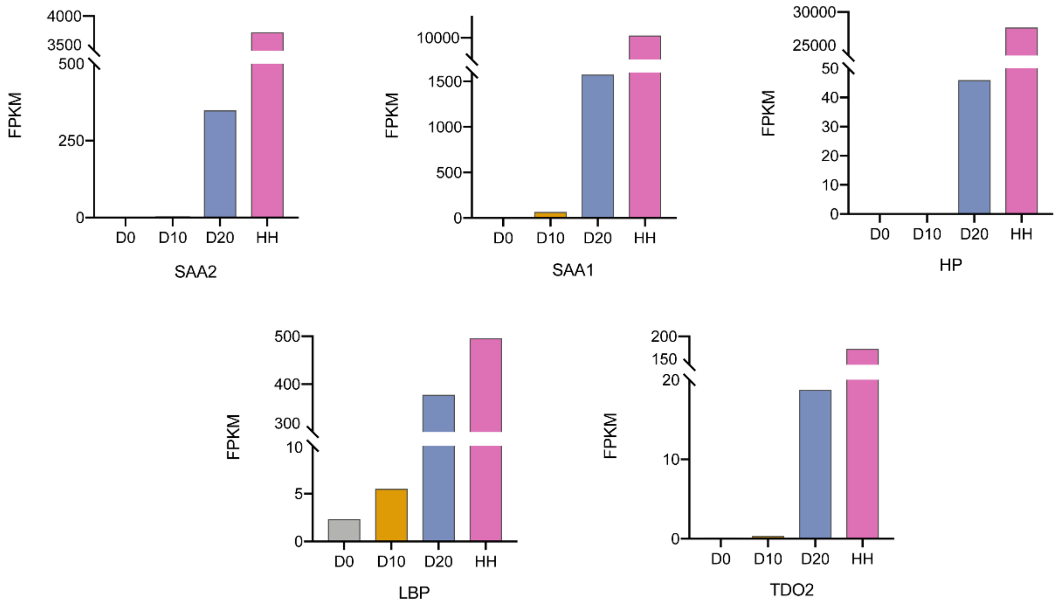

Supplement: Supplementary file 1 — Supplementary figure. [file ijmsv17p0263s1.pdf]
